# Antimicrobial susceptibility pattern of aerobic microbial isolates in a clinical laboratory in Karachi - Pakistan

**DOI:** 10.12669/pjms.293.3187

**Published:** 2013

**Authors:** Rubina Sabir, S. Faraz Danish Alvi, Asher Fawwad

**Affiliations:** 1 Rubina Sabir, Laboratory Manager, Clinical and Research Laboratory, Baqai Institute of Diabetology & Endocrinology, Baqai Medical University, Karachi, Pakistan.; 2 S. Faraz Danish Alvi, MBBS, Research Officer, Research Department, Baqai Institute of Diabetology & Endocrinology, Baqai Medical University, Karachi, Pakistan.; 3 Asher Fawwad, MBBS, M.Phil, Assistant Professor, Research Department, Baqai Institute of Diabetology & Endocrinology, Baqai Medical University, Karachi, Pakistan.

**Keywords:** Antimicrobial susceptibility, Multi-drug resistant (MDR), Microbial isolates

## Abstract

*Backgroun and Objective:* Resistance to multiple antimicrobials is the major cause of debility and death due to infectious diseases around the world. Our objective was to determine the frequency and antimicrobial susceptibility pattern of aerobic microbial isolates in a clinical laboratory.

***Methodology:*** All culture specimens of tissue, pus, urine, bone, blood, fluid, stool, sputum, and high vaginal swab received in the Microbiology Department of Clinical & Research Laboratory, Baqai Institute of Diabetology and Endocrinology from May 2010 to January 2011 were included in the present study. Bacterial isolates were identified and their antimicrobial susceptibility pattern was determined.

***Results:*** Out of 312 cultured specimens, 272 (87.17%) were found infected with 437 microbial organisms (412 bacteria and 25 Candida isolates). A total of 90 (20.59%) multi-drug resistant (MDR) isolates were found. MDR *Escherichia coli* was isolated in 40 (34.19%) out of 117 culture specimens which showed the growth of *Escherichia coli*, *Pseudomonas aeruginosa* in 17 (22.08%), Methicillin-resistant *Staphylococcus aureus* in 13 (11.50%), *Klebsiella pneumoniae* in 7 (22.58%), *Proteus *species in 6 (31.58%), *Acinetobacter* species in 3 (33.33%), *Enterobacter* species in 2 (28.57%), Coliform (*Escherichia coli*) in 1 (16.67%) and *Enterococcus *species were isolated in 1 (50%) culture specimen.

***Conclusions:*** High prevalence of multi-drug resistant bacteria was found in the present study. Emergence of antimicrobial resistance has become a major challenge in infectious disease medicine. Antimicrobial resistance may be due to misuse of antimicrobials by physicians and self medication in Pakistan. Further large scale studies are needed to validate our findings.

## INTRODUCTION

Infectious diseases remain a major cause of debility and death around the world and are responsible for worsening of living conditions of millions of people.^1^ Microbes (bacteria, fungi, parasites and viruses) cause infectious diseases and antimicrobial agents (such as penicillin, streptomycin and over 150 other antimicrobial) have been developed to combat the severity and spread of many of these diseases. The use of antimicrobial agents for prevention or treatment of infections in humans, animals and plants, in any dose and over any time period, cause a “selective pressure” on microbial populations.^2^ The emergence of resistance to antimicrobial in previously susceptible bacterial pathogens is a major challenge to infectious disease medicine.^3^

Antimicrobial resistance is a major public health concern in human medicine both in the community and in medical institutions.^4^ Antimicrobial resistance results in increased morbidity, mortality, and cost of health care. The rate of resistance varies in different studies.^5^ There was a high rate of nosocomial multi-drug resistant (MDR) organisms isolated from different specimen.^6^

Bacterial infections continue to be an important cause of morbidity and mortality in developing countries.^7^ During the last decade, an increase in the rates of antimicrobial resistance has been recognized worldwide and an increased frequency of MDR isolates in the clinical setting has been demonstrated.^8^

A study conducted in Ethiopia showed that 54.2% of eye swab cultures were positive for different bacterial pathogens.^9^ *Pseudomonas aeruginosa* found in urinary tract infections showed 19% multi-drug resistant strains in a German study.^10^ In a study in China, an opportunistic pathogen, *Acinetobacter baumannii* showed 30% drug resistance rate.^11^ Out of 165 samples received at Lahore Medical and Dental College in Lahore-Pakistan, 89 were found infected.^12^ While in a study on blood cultures, out of 1824 blood cultures, 508 (27.9%) yielded microorganism growth.^13^ In another study, the frequency of MDR *Pseudomonas aeruginosa* among all the *Pseudomonas aeruginosa* strains isolated was found to be 22.7%.^14^ Indiscriminate use of broad spectrum antimicrobial in hospital and community contributes to the emergence of infection with multi resistant organisms.^15^ There is an increase in the prevalence of MRSA among *S. aureus *isolates.^16^ *Pseudomonas aeruginosa *found an important cause of nosocomial and a multi drug resistant infections amongclinical isolates in local laboratory.^17^

Clinical laboratories play a pivotal role in providing accurate information and guidance in the treatment of microbial infections. In view of the above findings a study was conducted to determine the frequency and antimicrobial susceptibility patterns of microbial isolates in a clinical laboratory.

## METHODOLOGY

A descriptive study was conducted at the Microbiology Department of Clinical & Research Laboratory, Baqai Institute of Diabetology and Endocrinology (BIDE), a 24 hours laboratory service from May 2010 to January 2011. Laboratory records and data were used to study the pattern of multi-drug resistant microbial isolates.

***Inoculation of samples*** **: **Blood specimens received in blood culture bottles containing appropriate amount of blood in Tryptic Soy Broth were incubated for 24 hours or overnight at 35°C. After 24 hours these samples were sub cultured on blood agar, chocolate agar and MacConkey agar plates and incubated at 35°C overnight. Pus samples in syringe and swab were directly cultured on blood agar, chocolate agar, Sabouraud Dextrose agar and Mac-Conkey agar plates and incubated at 35°C overnight. Tissue and bone specimens received from foot clinic and Operation Theater in sterilized normal saline were crushed and then cultured on blood agar, chocolate agar and MacConkey agar plates and incubated at 35°C overnight. Identification of growth was based on colony morphology, gram staining and appropriate biochemical tests.^13^ Susceptibility to different antimicrobial, based on the type of growth was checked on Mueller Hinton agar by standard Kirby Bauer method.^13^

***Antimicrobial susceptibility test by disc diffusion method:*** The antimicrobial discs used for susceptibility testing were penicillin (10 μg), ampicillin (10 μg), amoxicillin (10 μg), clavulanic acid (30 μg), piperacillin/tazobactam (110 μg), oxacillin (1 μg), cephradine (30 μg), cefotaxime (30 μg), cefpirome (30 μg), cefuroxime (30 μg), vancomycin (30 μg), teicoplanin (30 μg), aztereonam (30 μg), imipenem (10 μg), meropenem (10 μg), amikacin (30 μg), gentamicin (10 μg), tobramycin (10 μg), erythromycin (15 μg), clarithromycin (15 μg), clindamycin (2 μg), nalidixic acid (30 μg), norfloxacin (10 μg), ofloxacin (5 μg), ciprofloxacin (5 μg), chloramphenicol (30 μg), sulphamethoxazole (25 μg), fosfomycin (50 μg), fusidic acid (10 μg) by OXOID shown in Table-I. Zone diameters to determine Antimicrobial susceptibility shown in Table-I.

***Multi-drug resistance: ***Multi-drug resistance was defined as organisms resistant to three or more drugs of the following class; Beta lactams (cefpirome, tazobactam), carbapenems (imepenem, meropenem), aminoglycosides (amikacin, gentamicin) and fluoroquinolones (ciprofloxacin).^18^

***Statistical Analysis: ***Data analysis was done on Statistical Package for Social Sciences (SPSS), version 13.0. Data presented in the form of frequency and percentage.

## RESULTS

A total of 312 specimens of diabetic foot tissue, pus, urine, bone, blood, fluid, stool, sputum, and high vaginal swab were received in the laboratory. On culture of these specimens, 40 showed no growth, while 272 (87.17%) specimens were found to have microbial pathogens. A total of 437 microbial organisms (412 bacteria and 25 Candida species) were isolated in 272 specimens.

**Table-I:** Antimicrobial Sensitivity Zone Pattern

*S#*	*Name*	*Disc* *Content*	*Sensitive*	*Intermediate*	*Resistant*
1	Clavulanic acid (AMC)	30 µg	≥ 18 mm	14 - 17 mm	≤ 13 mm
2	Piperacillin / Tazobactam (TZP)	10/100 µg	≥ 21 mm	18 - 20 mm	≤ 17 mm
3	Cefotaxime (CTX)	30 µg	≥ 26 mm	23 - 25 mm	≤ 22 mm
4	Vancomycin (VA)	30 µg	≥ 15 mm	-------	-------
5	Aztreonam (ATM)	30 µg	≥ 22 mm	16 - 21 mm	≤15 mm
6	Imipenem (IPM)	10 µg	≥ 16 mm	14 - 15 mm	≤ 13 mm
7	Meropenem (MEM)	10 µg	≥ 16 mm	14 - 15 mm	≤ 13 mm
8	Amikacin (AK)	30 µg	≥ 17 mm	15 - 16 mm	≤ 14 mm
9	Gentamicin (CN)	10 µg	≥ 15 mm	13 - 14 mm	≤ 12 mm
10	(Nalidixic acid) NA	30 µg	≥ 19 mm	13 - 18 mm	≤ 12 mm
11	(Norfloxacin) NOR	10 µg	≥ 17 mm	13 - 16 mm	≤ 12 mm
12	Ofloxacin (OFX)	05 µg	≥ 16 mm	---------	≤ 15 mm
13	Ciprofloxacin (CIP)	05 µg	≥ 21 mm	13 - 20 mm	≤ 12 mm
14	(Chloramphenicol) C	30 µg	≥ 18 mm	13 - 17 mm	≤ 12 mm
15	Sulphamethoxazole (SXT)	25 µg	≥ 16 mm	11 - 15 mm	≤ 10 mm
16	(Fosfomycin) FOS	200 µg	≥ 16 mm	13 - 15 mm	≤ 12 mm

**Table-III:** Frequency of multi-drug resistant (MDR) microbial isolates on culture

*Organism*	*n *	*MDR n (%)*
Escherichia coli	117	40 (34.19%)
Staphylococcus aureus	113	13 (11.50%)
Pseudomonas aeruginosa	77	17 (22.08%)
Klebsiella pneumoniae	31	7 (22.58%)
Candida species	20	------
Proteus species	19	6 (31.58%)
Acinetobacter species	9	3 (33.33%)
Mixed Growth	9	------
Streptococcus species	9	------
Enterobacter species	7	2 (28.57%)
Coliform	6	1 (16.67%)
Proteus vulgaris	6	------
Candida albicans	5	------
Proteus mirabilis	4	------
Enterococcus species	2	1 (50%)
Staphylococcus saprophyticus	2	------
Citrobacter species	1	------

Data presented as frequency and percentage

**Table-II:** Pattern of microbial isolates in culture specimen

*Specimen (n)*	*E coli (%)*	*S aureus (%)*	*P aeruginosa (%)*	*K pneumoniae (%)*	*Acinatobacter (%)*	*Proteus spp (%)*	*Candida spp (%)*	*Enterobacter spp (%)*	*Candida alb* *ican* *s (%)*	*Enterococcus spp (%)*	*P mirabilis (%)*	*Citrobacter spp (%)*	*Coliform (%)*	*P vulgaris (%)*	*Streptococcus spp (%)*	*S saprophyticus (%)*	*Mixed Insignif* *ican* *t growth (%)*	*Normal Flora (%)*	*No Growth (%)*
Tissue (99)	46 (46.46%)	42 (42.42%)	37 (37.3%)	16 (16.1%)	7 (7.0%)	5 (5.0%)	4 (4.0%)	4 (4.0%)	2 (2.0%)	2 (2.0%)	1 (1.0%)	1 (1.0%)	1 (1.0%)	1 (1.0%)	1 (1.0%)	-----	-----	--	--
Pus (98)	36 (36.73%)	48 (48.98%)	25 (25.5%)	7 (7.1%)	2(2.0%)	8 (8.1%)	9 (9.1%)	2(2.0%)	1 (0.2 %)	-----	-----	-----	3 (3.0%)	5 (5.1%)	5 (5.1%)	1 (0.2 %)	-----	--	--
Urine (55)	21 (38.18%)	6 (10.90%)		2 (3.6%)	-----	-----	6 (10.9%)	-----	2 (3.6%)	-----	-----	-----	2 (3.6%)				8 (14.5%)	--	--
Bone (34)	13(38.24%)	16(47.06%)	13(38.2%)	5 (14.7%)		6 (17.6%)	1 (2.9%)	1 (2.9%)		-----	2 (5.8%)	-----	-----	----	3 (8.8%)	1(2.9%)	-----	---	----
Blood (17)	1 (5.88%)	1 (5.88%)	1 (5.8%)	-----	-----	-----	-----	-----	-----	-----	-----	-----	-----	----	-----	-----	-----	----	14 (82.3%)
Fluid(6)	-----	-----	-----	-----	-----	-----	-----	-----	-----	-----	-----	-----	-----	-----	-----	-----	-----	----	6 (100%)
Stool (1)	-----	-----	-----	-----	-----	-----	-----	-----	-----	-----	-----	-----	-----	----	-----	-----	-----	1(1.8%)	----
Sputum (1)	-----	-----	1(1.8%)	-----	-----	-----	-----	-----	-----	-----	-----	-----	-----	----	-----	-----	-----	----	----
HVS (!)	-----	-----	-----	-----	-----	-----	-----	-----	-----	-----	-----	-----	-----	----	-----	-----	1(1.82%)	----	----

Data presented as frequency and percentage

 Pattern of microbial isolates in culture specimens are shown in Tables-II. On culture of 99 tissue specimens, 97 (97.97%) showed growth and overall 171 microbial organisms were isolated. *Escherichia coli* was the most frequent species, which isolated from tissue specimens (46:46.46%) specimens, followed by *Staphylococcus aureus* in (42:42.42%), and *Pseudomonas aeruginosa* was found in (37:37.37%). Among 98 pus specimens, 90 (91.83%) showed growth on culture and 152 organisms were isolated. *Staphylococcus aureus* was cultured from 48 (48.98%) and *Escherichia coli* from 36 (36.73%) samples. In 55 urine specimens, *Escherichia coli* was the most frequent isolated species (21:38.18%).

 All 34 bone culture specimens showed growth of microbial organisms, *Staphylococcus aureus* was isolated in 16 (47.06%) specimens. Details are given in Table-II.

A total of 90 (20.59%) multi-drug resistant organisms were found among 437 microbial isolates. *Escherichia coli* was isolated in 117 specimens, out of which 40 (34.19%) were found to be multi-drug resistant. Similarly, 17 (22.08%) isolated *Pseudomonas aeruginosa* in 77 culture specimens were multi-drug resistant. Out of 113 isolated *Staphylococcus aureus* in culture specimens, 13 (11.50%) were found to be multi-drug resistant as shown in Table-III.

## DISCUSSION

The present study shows local laboratory based data regarding the pattern of antibiotic susceptibility and MDR strains isolated from different clinical specimens. We found 87.17% of the cultured specimens infected with microbial pathogens, having a high prevalence of MDR strains (20.59%). There has been a major shift in the etiology of bacterial infections in the last decade. The more resistant pathogens have replaced the easily eradicable pathogens, thus leaving few options for choosing the right antimicrobial for treatment.^19^

In a study conducted in urinary tract infection (UTI) patients, 90% of the gram negative isolates were found to be MDR.^20^ In another study on fecal isolates, the frequency of MDR bacteria was markedly high (31.7%)^21^, while in a study on diabetic foot ulcer patients, 45% were found to be MDR.^22^ Similarly in another study, 77.3% of bacterial isolates in ocular infections were MDR to the commonly prescribed antimicrobial.^9^ However, in a local study 78% of gram negative bacteria were found to be MDR.^23^

The most common isolates observed in our study were *Escherichia coli*, *Staphylococcus aureus, Pseudomonas aeruginosa, Klebsiella pneumoniae*, Candida and *Proteus* species. Similar findings were observed in another study.^18^ The most frequent isolate in our study was *Escherichia coli*, which was isolated in 26.77% of the culture specimens, while in other studies it was isolated in 47.4%9, 41.7%^24^ and 32.39%^25^ of the cultures. In a study on combat-injured personnel during Operations Iraqi Freedom and Enduring Freedom 57% of the *Escherichia coli* were MDR^26^, while in our study 34.19% were found to be MDR. Our study findings are supported by other studies.^24^^,^^25^

Methicillin resistant *Staphylococcus aureus* (MRSA) has become an enormous problem for health care providers, it is hard to treat and is sometimes called “super bug”. The prevalence of *Staphylococcus aureus* isolation was 25.86% in our study while in another study it was 15.49%.^27^ In our study 11.50% of the total MRSA isolates were found while in other studies the frequency of MRSA were 23.9%^28^ and 22.9%.^29^

In this study we found 22.08% MDR strains of *Pseudomonas aeruginosa* while in another local study it was found to be 22.7%^14^, similar to the findings of our study. In a study conducted in acute and chronic UTI patients, 19% multi-drug resistant *Pseudomonas aeruginosa* were isolated^10^ while in another study in intensive car (ICU) and non-intensive care unit (ICU) patients there were 3% MDR *Pseudomonas aeruginosa* in 2004 and 2% in 2005 and MDR *Pseudomonas aeruginosa *was more commonly found in non-intensive care unit (ICU) patients.^30^

Frequency of MDR *Klebsiella pneumoniae* in our study was 22.58%, while in other study it was found to be 11.0%.^21^ Amongst the gram negative bacteria the most common organism was *Klebsiella* Species 24.1%.^13^

In a New York Hospital study the majority, 70% of the isolates were multi-drug resistant in *Acinetobacter baumannii*^31^, while 33.33% MDR *Acinetobacter* species were found in our study.

## CONCLUSIONS

High prevalence of multi-drug resistant bacteria was found in the present study. Emergence of antimicrobial resistance has become a major challenge in infectious disease medicine. Self and improper medication is a very serious and common issue is Pakistan which play an important role in antimicrobial resistance. Along with self medication misuse of antimicrobials by physicians also makes the situation verse. Further large scale studies are needed to validate our findings.
